# The face of Ebola: changing frequency of haemorrhage in the West African compared with Eastern-Central African outbreaks

**DOI:** 10.1186/s12879-015-1302-4

**Published:** 2015-12-11

**Authors:** Stefano Petti, Giuseppe Alessio Messano, Enzo Maria Vingolo, Luigi Tonino Marsella, Crispian Scully

**Affiliations:** Department of Public Health and Infectious Diseases, Sapienza University, Piazzale Aldo Moro 5, 00185 Rome, Italy; Ophthalmology Department, Sapienza University, Viale del Policlinico 155, 00186 Rome, Italy; Department of Biomedicine and Prevention, Tor Vergata University, Viale Oxford 81, 00133 Rome, Italy; University College London, Gower Street WC1E 6BT, London, UK

**Keywords:** Ebolavirus disease, Case-fatality rate, Haemorrhage, Conjunctivitis, Epistaxis, Gingival bleeding

## Abstract

**Background:**

The West-African (WA) Zaire Ebolavirus disease (EVD) outbreak was characterized by an exceptionally high number of cases and deaths as compared with the Eastern-Central African (ECA) outbreaks. Despite the Zaire Ebolavirus being the most lethal for humans, case-fatality rate, close to 80 % in ECA outbreaks, almost halved to 47 % in Guinea-Liberia-Sierra Leone (WA). Such an improvement was due to the remarkable implementation of international humanitarian aids. Some studies also suggested that the long human-to-human transmission cycle occurred in WA, gave rise to human adaptation and consequent immune escape. Haemorrhage, the main feature in seriously infected EVD patients, is due to the immune system that triggers the infected endothelial cells which expose the spike-like glycoprotein (GP) of the virion on their surface. If the human adaptation hypothesis holds true, the proportion of EVD patients with haemorrhage in the WA outbreak should be lower than in the ECA outbreaks due to immune escape. Therefore, the aim of this meta-analysis was to compare the relative frequencies of three typical haemorrhagic symptoms (conjunctival –CB, nasal –NB, gingival –GB- bleedings) in the ECA and WA outbreaks.

**Methods:**

Literature searches were performed through PubMed and Scopus using generic keywords; surveys including at least ten patients reporting CB, NB, GB relative frequencies were extracted and split into ECA and WA. The meta-analytical methods chosen were based on the levels of between-study heterogeneity and publication bias. Pooled CB, NB, GB relative frequencies in ECA and WA were estimated and compared. Subgroup analysis including only studies on Zaire Ebolavirus also was performed.

**Results:**

Fifteen studies (10 ECA, 5 WA) were located with 4,867 (CB), 3,859 (NB), 4,278 (GB) EVD patients overall. GB pooled relative frequency was 45.3 % (95 % confidence interval -95 CI, 34.7–56.1 %) and 18.0 % (95 CI, 6.0–34.5 %), in ECA and WA; NB was 10.6 % (95 CI, 5.7–16.8 %) and 1.3 % (1.0–1.8 %); GB was 24.2 % (95 CI, 11.9–39.2 %) and 1.9 % (95 CI, 1.4–2.4 %). Subgroup analysis confirmed these results.

**Conclusions:**

During the WA outbreak the relative frequency of GB decreased by two thirds, while NB and GB almost disappeared, suggesting that the Zaire Ebolavirus human adaptation hypothesis is plausible.

**Electronic supplementary material:**

The online version of this article (doi:10.1186/s12879-015-1302-4) contains supplementary material, which is available to authorized users.

## Background

Ebolavirus disease (EVD) is a zoonosis with persistence of the virus in reservoir species. Indeed, at least three species of fruit bats (suborder *Megachiroptera*) are naturally infected: *Hypsignathus monstrosus* (hammer-headed fruit bat), *Epomops franqueti* (singing fruit bat) and *Myonycteris torquata* (little collared fruit bat). Asymptomatically-infected bats drop partially eaten fruit and masticated fruit pulp, contaminated by their saliva and blood, to the ground, where they are eaten by great apes and forest duikers (antelopes). Ebolavirus may then be transmitted from these infected animals to humans. Humans and primates have been considered the end hosts and have not been regarded as reservoir species [1]. Ebolavirus is transmitted between humans through direct contact with blood or blood-containing vomit, faeces and other bodily fluids (or tissues) from EVD patients in the acute stage of the disease. Conversely, subjects who reside in confined, shared and close spaces, but who have no direct physical contact with acute cases, do not develop EVD [2–4]. Transmission through air is unlikely [5, 6], while sexual transmission through infected patients is possible [7]. EVD has an incubation period of 2–21 days and the main symptoms are fever, headache, fatigue, myalgia, diarrhoea, vomiting, abdominal pain and, typically, bleeding including conjunctival, nasal, gingival bleeding and bleeding from skin and injection sites [8, 9]. Recovery may be followed by muscle pains and prolonged virus carriage.

The first cases of EVD were reported in 1976 in southern South Sudan and in the northern Democratic Republic of the Congo [10, 11]. Since 1976, human-to-human transmission has been reported in several Eastern-Central African outbreaks [12]. The latest EVD outbreak started in Guinea (West Africa), the index case was a 18-month-old boy died in December 2013, but the outbreak was not detected until March 2014, thus leaving thousands of people becoming infected. In this largest outbreak ever seen, the case number was 100–1000 times greater than in previous outbreaks [13]. Assuming that the EVD attack rate did not change significantly from previous outbreaks [11, 14–16] and that in the early stages of the West African outbreak the Ebolavirus did not undergo important genetic changes affecting transmission rate [17–19], such an increase was probably explained by the exceptional human crowding due to both the urban setting and the generally higher population density in West Africa than in Eastern-Central Africa [20], which led to a large increase in the number of exposed individuals. In addition to human crowding there were other social determinants explaining the West-African outbreak, which, according to the World Health Organization (WHO), were: (1) Extremely damaged Public Health infrastructures in Guinea, Liberia and Sierra Leone, which only recently emerged from years of civil war; (2) High population mobility across uncontrolled borders, another consequence of war; (3) Severe shortages of trained healthcare workers: before the 2013–2015 outbreak there were 1–2 doctors every 100,000 individuals; (4) Cultural beliefs and behavioral practices, such as the adherence to ancestral funeral and burial practices. The WHO has estimated that 60-80 % EVD cases were attributable to these practices; (5) Reliance on traditional healers: as the outbreak began, the high EVD mortality in healthcare facilities inducing local people to think that hospitals were the places where contagion and death actually occur; (6) Community resistance: many people refused to believe that EVD was real because they and their ancestors had been living in the same environment and had never developed EVD before; (7) Public Health messages that were intended to promote protective behaviors proved to have the opposite effect, because they emphasized that the disease was extremely serious and there was no vaccine or treatment; (8) Other endemic infectious diseases, such as cholera and malaria, which can be difficult to distinguish from EVD in the early stages; (9) Ebolavirus is endemic in West Africa; and (10) Spread by international air travel [21].

Genome surveillance studies revealed that while during the Eastern-Central African outbreaks the Ebolavirus evolution rate was 0.5-8 × 10^−4^ nucleotide substitutions per site per year [22], in West Africa such a rate raised to 9–20 × 10^−4^ nucleotide substitutions per site per year [17, 22–25] showing an accelerated evolution. In addition, many nonsynonymous nucleotide substitutions occurred on the surface of the spike-like glycoprotein (GP; which plays an important role in the virus entry into cells) [26] and promoted immune evasion [27].

These data support the hypothesis that after a series of tragic coincidences due to environmental factors, which promoted the spread of the outbreak, the persistence of the human-to-human transmission cycle gave raise to genetic changes which caused the generation of several lineages and to human adaptation [27]. This hypothesis is corroborated by the facts that similar trends have been observed for other cell receptor binding enzymes, such as the neuraminidase of the influenza virus subtypes A H1N1 and A H3N2 [28] and that a similar intra-individual evolution occurs to the envelope glycoprotein of hepatitis C virus after the acute phase of the infection to escape from the host immune response and establish persistent (i.e., chronic) infection [29, 30].

Another element in support of this hypothesis is the change in case-fatality rate. Indeed, Zaire Ebolavirus is the most lethal species with an estimated case-fatality rate of 76 %, considerably higher than the Sudan (55 %) and the Bundibugyo (37 %) species [31]. During the latest Zaire Ebolavirus outbreak the case-fatality rate dropped to 47 % (considering only confirmed cases and deaths in Guinea and Sierra Leone) [13]. Such a decrease in case-fatality rate is typical of human adaptation [28] and is in line with the theory that the majority of human pathogens arose only after the advent of agriculture and originate from animal pathogens: they were initially responsible for zoonoses and later adapted to the human hosts, due to co-habitation between animals and humans and to human crowding [32]. The reported decrease in EVD case-fatality rate during the West-African EVD outbreak is, however, principally due to the prompt implementation of control and therapeutic measures by local and international organizations from all over the world (see, https://en.wikipedia.org/wiki/Responses_to_the_Ebola_virus_epidemic_in_West_Africa for review. Accessed August 24, 2015).

Haemorrhage is a serious EVD consequence, often responsible for patients' demise. Actually, during the first EVD outbreak in South Sudan, almost all (91 %) of the fatal cases and only one half (48 %) of the nonfatal cases showed some visible bleeding features [10]. This link between the onset of haemorrhage and death is due to the fact that Ebolavirus primarily infects endothelial cells. GP is responsible for virus internalization and is then displayed on the external surface of the infected cells. Haemorrhage is, therefore, due the lysis of infected endothelial cells caused by the human immune system triggered by GP [33].

Therefore, given the association between death and haemorrhage in EVD patients, in order to evaluate whether the large decrease in case-fatality rate observed during the West African outbreak was exclusively due to humanitarian aids or could be explained by Ebolavirus human adaptation, the current study sought to investigate the relative frequency of EVD patients with haemorrhagic features. More specifically, if the immune evasion hypothesis holds true, patients with haemorrhage should be less frequent during the West-African outbreak than during the Eastern-Central African outbreaks. If, alternatively, Ebolavirus adaptation to humans did not occur in West Africa, the frequency of EVD patients with haemorrhage did not vary. Thus, this meta-analysis sought to estimate the pooled relative frequencies of conjunctival and orofacial (nasal, gingival) haemorrhagic symptoms observed during the earlier Eastern-Central African and the latest West African outbreaks.

## Methods

A meta-analysis was performed to estimate the pooled relative frequency of the three main orofacial haemorrhagic symptoms/signs, that is, conjunctival bleeding/injection (and conjunctivitis), epistaxis and gingival bleeding, features chosen because they are easily detectable, although they are not exclusive of EVD. It was assumed that the other conditions and diseases associated with similar symptoms (e.g. gingivitis) did not change in frequency within the study populations throughout these years and, therefore, did not interfere with the present analysis.

Literature searches were performed through Scopus and PubMed without time and language restrictions. Inclusion criteria were surveys reporting conjunctival, nasal and/or gingival bleeding in samples of at least ten EVD patients. Exclusion criteria were all non-relevant studies (e.g., studies on animals, laboratory studies, reviews, etc.), case reports/samples of less than ten EVD patients, surveys which did not report bleeding features analytically.

Generic key words were used to minimize selection bias which, in meta-analyses, refers to undetected published studies. Selection bias was considered more pervasive than publication bias, which refers to unpublished studies, due to the recent explosion of scientific journals and publications [34]. Thus, key words used were “Ebola” (title, abstract, key words) AND “symptom” (all fields).

Titles and abstracts were used to exclude non-relevant studies. Full texts of remaining surveys were searched and read and only surveys which met the inclusion criteria were selected. The reviewers independently extracted conjunctival bleeding (EMV), epistaxis (LTM) and gingival bleeding (GAM) relative frequencies. Extracted data were then supervised and meta-analyzed by the two other authors (SP and CS).

The pooled relative frequencies of the three features were assessed. The meta-analytic method was chosen on the basis of the level of between-study heterogeneity, which was investigated with the I^2^ statistic [35]. For non-significant I^2^ values the meta-analysis was performed with the fixed-effects method, for significant I^2^ values the random-effects method was used.

Publication bias was formally investigated [36] with the test of Egger and colleagues [37], if the test was significant the number of studies was adjusted for publication bias using the trim and fill method and identifying the potentially missing studies with the R_0_ method [38, 39].

Sensitivity analysis to study inclusion was made to investigate whether the pooled relative frequency estimates were influenced by a single study.

Surveys were then split according to the country where they have been performed into Eastern-Central African and West African outbreaks. Pooled relative frequency estimates of conjunctival nasal and gingival bleeding were assessed for these subgroups using the aforementioned methodology.

Studies on conjunctival bleeding were split into studies which reported bleeding or injected eyes (conjunctival bleeding) and studies which reported more generic conjunctivitis. Pooled relative frequency estimates were assessed for all studies together, for Eastern-Central African studies and for West African studies.

Subgroup analysis also was performed to investigate whether there were differences between Zaire Ebolavirus outbreaks. Therefore, since the West-African outbreak was exclusively due to the Zaire Ebolavirus species, surveys from Eastern-Central Africa reporting only Zaire Ebolavirus outbreaks were considered and the pooled relative frequency estimates of conjunctival nasal and gingival bleeding were assessed.

This article followed the MOOSE guidelines for reporting meta-analyses of observational studies [40]. The statistical analyses were performed using StatView 5.0.1 (SAS Institute Inc., NC,US). The level of significance was set at 95 %.

## Results

**Fig. 1:**
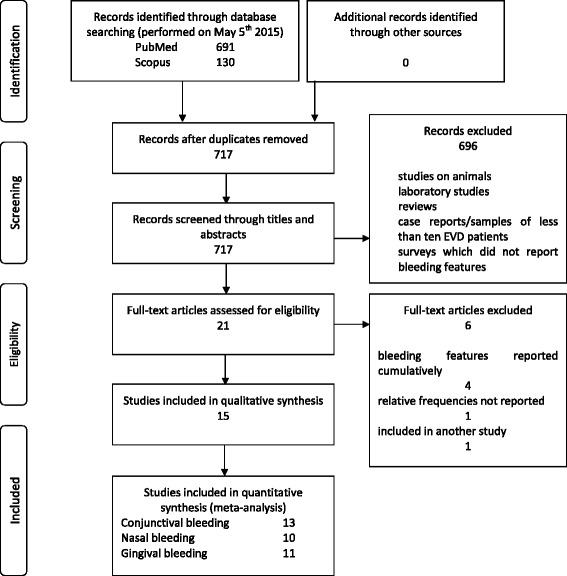
PRISMA 2009 flow diagram of primary study selection procedure

The Scopus and PubMed searches, performed on October 15, 2015, provided 130 and 691 studies, respectively (Fig. 1). Most of the studies did not fall into the inclusion criteria, thus leaving twenty-one eligible studies. Four of these were excluded because bleeding features were reported cumulatively [10, 41–43], one because the relative frequency was not reported [44], another one because it was part of a larger sample [45], thus fifteen primary studies were available for meta-analysis [2, 11, 46–58]. All studies were performed during Zaire Ebolavirus outbreaks, excluding two studies performed during Sudan Ebolavirus [2, 51] and one study during a Bundibugyo Ebolavirus [52] (Table 1) outbreak. Five studies were performed during the West African outbreak [54–58], the remainder during the Eastern-Central African outbreaks.

**Table 1:** Characteristics of the outbreaks described by the primary studies

Study	Ebolavirus	Country	Survey year
WHO, [11]	Zaire	Democratic Republic of Congo	1976
Baron et al., [2]	Sudan	South Sudan	1979
Sureau, [46]	Zaire	Democratic Republic of Congo	1976
Bwaka et al., [47]	Zaire	Democratic Republic of Congo	1995
Georges et al., [48]	Zaire	Gabon	1996
Khan et al., [49]	Zaire	Democratic Republic of Congo	1995
Ndambi et al., [50]	Zaire	Democratic Republic of Congo	1995
Mupere et al., [51]	Sudan	Uganda	2000
Roddy et al., [52]	Bundibugyo	Uganda	2007
Maganga et al., [53]	Zaire	Democratic Republic of Congo	2014
Schieffelin et al., [54]	Zaire	Sierra Leone	2014
Bah et al., [55]	Zaire	Guinea	2014
Dallatomasina et al., [56]	Zaire	Sierra Leone	2014
WHO Ebola Response Team, [57]	Zaire	Guinea, Liberia, Sierra Leone	2014
Yan et al., [58]	Zaire	Sierra Leone	2014

The number of EVD patients considered for conjunctival bleeding were 4,867 (677 from Eastern-Central Africa, 4,190 from West Africa), those considered for nasal bleeding were 3,859 (522 from Eastern-Central Africa, 3,337 from West Africa), those considered for gingival bleeding were 4,278 (972 from Eastern-Central Africa, 3,306 from West Africa) (Additional file 1: Table S1). The relative frequencies of conjunctival nasal and gingival bleeding reported by the primary studies are displayed in Table 2. Conjunctival bleeding was reported by thirteen studies, epistaxis by ten studies, gingival bleeding by eleven studies.

**Table 2:** Relative frequencies (95 % confidence intervals between parentheses) of conjunctival, nasal and gingival bleeding features reported by the primary studies

Study	Conjunctival bleeding and conjunctivitis	Nasal bleeding	Gingival bleeding
WHO, [11]	55.0 % (48.5–61.3 %)		67.4 % (61.4–73.3 %)
Baron et al., [2]		21.9 % (7.6–36.2 %)	34.4 % (17.9–50.8 %)
Sureau, [46]		14.7 % (10.5–19.0 %)	22.3 % (17.3–27.3 %)
Bwaka et al., [47]	42.7 % (33.0–52.8 %)	1.9 % (0.0–4.6 %)	12.6 % (6.2–19.0 %)
Georges et al., [48]	53.3 % (26.6–78.7 %)	13.3 % (0.0–30.5 %)	40.0 % (15.2–64.8 %)
Khan et al., [49]	35.7 % (29.2–42.6 %)		22.1 % (16.5–27.8 %)
Ndambi et al., [50]	78.3 % (56.3–92.5 %)	4.3 % (0.0–12.7 %)	30.4 % (11.6–49.2 %)
Mupere et al., [51]	40.0 % (19.1–63.9 %)	10.0 % (0.0–23.1 %)	10.0 % (0.0–23.1 %)
Roddy et al., [52]	50.0 % (29.9–70.1 %)	7.7 % (0.0–17.9 %)	3.8 % (0.0–11.2 %)
Maganga et al., [53]	15.8 % (6.0–31.3 %)	10.5 % (0.8–20.3 %)	7.9 % (0.0–16.5 %)
Schieffelin et al., [54]	25.0 % (13.2–40.3 %)		
Bah et al., [55]	10.8 % (3.0–25.4 %)	5.4 % (0.0–12.7 %)	
Dallatomasina et al., [56]	2.0 % (0.7–4.7 %)		
WHO Ebola Response Team, [57]	26.0 % (24.6–27.4 %)	1.3 % (0.9–1.7 %)	1.9 % (1.4–2.4 %)
Yan et al., [58]	34.3 % (25.4–44.0 %)		

Between-study heterogeneity was significantly high for all these features (Table 3). Therefore, the random-effects method was used. According to the test of Egger and colleagues, the degree of publication bias was not significantly high and no adjustment was performed (data not in Table). The Forest plots (Additional file 2: Figure S1) including all studies show that relative frequency of conjunctival bleeding apparently decreased from the oldest to the newest study (Additional file 2: Figure S1a), the same trends were observed for epistaxis (Additional file 2: Figure S1b) and gingival bleeding (Additional file 2: Figure S1c). The pooled relative frequency estimates showed that conjunctival bleeding/conjunctivitis was the most frequent feature and was reported in 34 % (95 % confidence interval, 23–45 %) EVD patients, followed by gingival bleeding (21 %; 95 % confidence interval, 7–40 %) and epistaxis (9 %; 95 % confidence interval 3–16 %). Conjunctival bleeding/conjunctivitis was significantly more frequent than epistaxis, while the differences between the other features were not significant (Table 3). Sensitivity analysis to exclusion criteria corroborated these estimates, since none of the studies, excluded in turn, produced a statistically significant departure from the estimates obtained with all the primary studies included (Additional file 3: Table S2). The difference between conjunctivitis and conjunctival bleeding/injection was non-significant: 36 % (95 % confidence interval, 21–52 %) and 31 % (95 % confidence interval 21–42 %).

**Table 3:** Pooled relative frequency estimates of conjunctival, nasal and gingival bleeding features. Between–study heterogeneity (I^2^ statistic, 95 % confidence interval)

Bleeding	Pooled relative frequency	95 % confidence interval	I^2^ statistic
Conjunctival (all)	33.6 %	23.5–44.6 %	94.6–97.2 %
Conjunctivitis only	35.9 %	20.9–52.5 %	96.4–98.3 %
Conjunctival bleeding/injection	31.1 %	21.3–41.8 %	42.1–90.3 %
Nasal	8.7 %	3.5–16.0 %	87.8–95.1 %
Gingival	21.1 %	7.0–40.1 %	98.4–99.0 %

Studies performed during the Eastern-Central African outbreaks were eight for conjunctival bleeding [10, 47–53] and epistaxis [2, 46–48, 50–53], ten for gingival bleeding [2, 10, 46–53], while studies performed during the West African outbreak were five for conjunctival bleeding [54–58], two for epistaxis [55, 57] and one for gingival bleeding [57]. The random-effects method was used in almost all cases. The Forest plots including only Eastern-Central African studies were regularly distributed across the pooled estimates (Additional file 4: Figure S2). Fairly irregular distributions were observed for West African studies, due to their limited number. Conjunctival nasal and gingival bleeding features were significantly more frequent in the Eastern-Central African studies (Table 4). Conjunctival bleeding pooled relative frequency was 45 % in Eastern-Central Africa (95 % confidence interval, 35–56 %) and 18 % in West Africa (95 % confidence interval, 6–34 %); epistaxis was 11 % in Eastern-Central Africa (95 % confidence interval, 6–17 %) and only 1 % in West Africa (95 % confidence interval, 1–2 %); gingival bleeding was 24 % in Eastern-Central Africa (95 % confidence interval, 12–39 %) and only 2 % in West Africa (95 % confidence interval, 1–2 %).

**Table 4:** Pooled relative frequency estimates of conjunctival, nasal and gingival bleeding features in the Eastern–Central African outbreaks (all outbreaks, Zaire Ebolavirus outbreaks) and in the West–African outbreak. Between–study heterogeneity (I^2^ statistic, 95 % confidence interval)

	Pooled relative frequency	95 % confidence interval	I^2^ statistic
Conjunctival bleeding/injection and conjunctivitis			
Eastern–Central Africa (8 studies)	45.3 %	34.7–56.1 %	70.7–91.6 %
Eastern–Central Africa (Zaire Ebolavirus, 6 studies)	45.4 %	32.6–58.4 %	77.9–94.2 %
West Africa (5 studies)	18.0 %	6.0–34.5 %	95.6–98.3 %
Conjunctival bleeding/injection only			
Eastern–Central Africa (3 studies)	38.2 %	33.0–43.6 %	0.0–95.5^a^
West Africa (2 studies)	18.8 %	11.0–28.8 %	0.0–91.3^a^
Conjunctivitis only			
Eastern–Central Africa (5 studies)	50.0 %	31.0–68.2 %	72.0–94.0 %
West Africa (3 studies)	17.9 %	3.0–41.5 %	95.6–99.2 %
Nasal bleeding			
Eastern–Central Africa (8 studies)	10.6 %	5.7–16.8 %	33.1–84.8 %
Eastern–Central Africa (Zaire Ebolavirus, 5 studies)	9.0 %	3.3–17.2 %	47.7–90.9 %
West Africa (2 studies)	1.3 %	1.0–1.8 %	0.0–92.8 %^a^
Gingival bleeding			
Eastern–Central Africa (10 studies)	24.2 %	11.9–39.2 %	93.6–97.0 %
Eastern–Central Africa (Zaire Ebolavirus, 7 studies)	27.9 %	12.5–46.7 %	95.1–97.9 %
West Africa (1 study)	1.9 %	1.4–2.4 %	Not applicable^b^

^a^non–significant I^2^statistic, fixed–effects meta–analytic method; ^b^prevalence estimate reported by a single study

All the differences between pooled relative frequencies in Eastern–Central Africa and West Africa are statistically significant at 95 % level

Eastern-Central African studies focusing on Zaire Ebolavirus outbreaks were six for conjunctival bleeding [10, 47–50, 53], five for epistaxis [46–48, 50, 53], seven for gingival bleeding [10, 46–50, 53]. The random-effects method was used due to large between-study heterogeneity (Table 4). No adjustment for publication bias was performed because of regular distribution of the studies in the Forest plots (data not shown). Pooled relative frequencies of conjunctival, nasal and gingival bleeding were 45 % (95 % confidence interval, 33–58 %), 9.0 % (95 % confidence interval, 3–17 %), 28 % (95 % confidence interval, 12–47 %), respectively. Differences with the West African outbreak were significant at 95 % level.

## Discussion

One problem in assessing the relative frequency of EVD haemorrhagic signs is that features were reported by healthcare workers when the patients presented at the healthcare facilities and therefore, depend on the disease stage at presentation (which, in turn, depends on the level of the population awareness toward EVD) and on the reporting protocol adopted by the healthcare staff. Although there are no elements to support this hypothesis, it is reasonable to conjecture that if the general population was unaware of EVD features, infected subjects with epistaxis, gingival bleeding and/or conjunctival bleeding as main features were less likely to present to healthcare facilities than were subjects more obviously ill with high fever, vomiting and/or diarrhoea. Few of the healthcare workers would be trained specifically in ENT/Ophthalmology and/or Dental examinations, and facilities for these were likely uncommon. Thus, primary studies must yield some degree of reporting bias, which probably led to underestimate the true relative frequencies of the these bleeding features. However, if reporting bias was present in the African healthcare facilities, it was probably lower in the studies used for this meta-analysis, because authors often followed the WHO EVD case definition recommendations, which comprised gingival bleeding and conjunctivitis among the key features [59].

Another issue with the present meta-analysis was selection bias, which usually refers to published studies that have not been located. In this analysis it may also refer to the inclusion of partially duplicate studies, which reported data from the same sets of patients and to the exclusion of studies which were mistakenly considered duplicate and actually were original. This problem regards principally the West African outbreak, due to the exceptionally high number of published studies. In order to obtain the most reliable pooled estimates, the risk to exclude potentially original studies was preferred to the risk to reuse data from the same set of patients. Indeed, in the first case, the limited number of patients led to broader confidence intervals, while in the latter case, the inclusion of duplicate data may have raised the burden of the bias of the primary study, in the event that there was any.

The present analysis suggests that conjunctival bleeding is the most frequent EVD orofacial symptom. However, it is not possible to diagnose EVD solely on the basis of conjunctival bleeding even if fever, vomiting and diarrhoea are co-existing, because these features are frequently present in many infectious diseases common in Eastern-Central and West Africa, such as measles [60] and malaria [61]. Very interestingly, conjunctival bleeding/injection along with conjunctivitis, which were detected in almost one half EVD patients during the Eastern-Central African outbreaks, were reported in less than one every six patients during the West African outbreak. As for the two other investigated features, they were detected relatively frequently during Eastern-Central African outbreaks, that is, one every four patients (gingival bleeding) and one every ten patients (epistaxis), while they were almost non-existent during the West African outbreak, being detectable, at the best, in only one in every forty to fifty EVD patients. These data are corroborated by another survey which reported that there were only three subjects with unexplained bleeding in a sample of 103 EVD patients in Sierra Leone [58].

Such a huge decrease in ocular and orofacial EVD features, which occurred during the West African outbreak, parallels the significant decrease in case-fatality rate, which fell by one third, from 65 % (95 % confidence interval, 55–75 %) during the Eastern-Central African outbreaks [31], to 47.0 % (95 % confidence interval, 46.2–47.9 %) in the West African outbreak –estimated using data from Guinea and Sierra Leone reported by the WHO [13]. In addition, during the West African outbreak, the case-fatality rate progressively decreased in rural areas starting from rates similar as those observed in previous EVD outbreaks. This decrease is in line with the hypothesis of the adaptation of Ebolavirus to the human host [27], and is not in contrast with the indisputable excellent results due to the implementation of control policies, such as the national strategy for the Rapid Isolation and Treatment of Ebola (RITE) in Liberia [62].

Another element supporting these results showing that haemorrhagic features drastically decreased during the West African outbreak, is that in 20 % EVD patients the transmission route was unexplained [4]. This suggests that in one fifth of transmissions, the signs and the symptoms of EVD subjects who acted as infection source were so slight that the infected person could not remember the transmission event.

An element of debate is the meaning of the doubled mutation rate of the Ebolavirus occurred during the West African outbreak. According to two studies performed on whole Zaire Ebolavirus genome and on GP glycoprotein sequence, there were no signs of positive selection, which would have definitively confirmed the human adaptation hypothesis [18, 19]. Conversely, most studies considered as evident signs of selective pressure events such as the emergence of multiple, even intra-host, novel Ebolavirus lineages during the West African outbreak, the many non-synonymous RNA nucleotide changes leading to amino-acid changes, some of them at positions of high level of conservation across Eastern-Central African Ebolaviruses and the positive selection within the GP glycoprotein amino-acidic sequence, which was not reported during previous outbreaks [17, 23–25, 30, 63].

These data suggest that, although the above-mentioned social determinants have been responsible for part of the reduced case-fatality reported during the West-African EVD outbreak, human adaptation, along with immune evasion, may have occurred. Indeed, in the classification of stages leading to endemic human pathogens from animal pathogens, Ebolavirus was previously classified at stage 3, that is, an animal pathogen that can undergo only a few cycles of secondary transmission between humans, so that occasional human outbreaks triggered by a primary infection soon die out. However, the West African outbreak yielded the characteristics of a stage-4 pathogen, that is, an infectious disease that exists in animals and that infects humans cyclically by primary transmission from animal host, but that also undergoes long sequences of secondary transmission between humans, without the involvement of animal hosts. Examples of stage-4 infectious diseases are typhus, yellow fever and influenza A [32]. According to this theory of the animal origin of human pathogens, transitions from stage 3 to stage 4 frequently occurred during human evolution. However, the major barrier of such a transition is that animal pathogens need long secondary transmission chains to evolve adaptation to the novel host. This condition was prevented in the case of EVD by the small size of human communities where the outbreaks occurred. Indeed, Eastern/Central African outbreaks took place in villages where the number of susceptible hosts rapidly ended up, because susceptible individuals died or became immune, as demonstrated by the high case-fatality rate –as high as 80–90 %- and the high immunization rate –as high as 30 %, in villages where EVD outbreaks occurred [2]. However, according to this theory, some external factors, such as frequent blood exposures, presence of susceptible pools of immunosuppressed hosts (e.g., malnourished populations) and large population sizes, may intervene to protract human-to-human transmission chains, thus promoting the transition from stage 3 to stage 4 [32].

In line with this theory and accounting for the reported decreased proportion of EVD patients with haemorrhagic symptoms, it is possible to conjecture that the aforementioned West Africa-specific social determinants have been responsible for the initially high number of secondary (and undetected) transmissions between humans [21]. Thanks to this long human-to-human transmission cycle [10] the Ebolavirus had the opportunity to adapt to the human host.

## Conclusions

In conclusion, along with the increased Ebolavirus mutation rate and the decreased case-fatality rate that occurred during the West African outbreak, this outbreak was also characterized by a significant decrease in the proportion of patients with bleeding features. Some of these features, that is, nasal and gingival bleeding almost disappeared. These data support the hypothesis of Ebolavirus adaptation to human host.

## Additional files

Additional file 1: Table S1. Number of EVD patients (note that in some studies the number of patients considered for the three bleeding features were different, suggesting that in some cases the clinical examination protocol was not the same within the study). (DOCX 14 kb) Additional file 2: Figure S1. Forest plots of the meta-analyses of the relative frequencies of conjunctival (a), nasal (b) and gingival (c) bleeding among EVD patients. Note the differences between Central and West African (Schieffelin et al., [54]; Bah et al., [55]; Dallatomasina et al., [56]; WHO, 2015 [57]; Yan et al., [58]) studies. (DOCX 27 kb) Additional file 3: Table S2. Sensitivity analysis to study inclusion. One study was excluded in turn and the pooled relative frequencies were re-estimated with the random-effects method. (DOCX 14 kb) Additional file 4: Figure S2. Forest plots of the meta-analyses of the relative frequencies of conjunctival (Central African studies –a, West African studies –b), nasal (Central African studies –c, West African studies –d) and gingival bleeding (Central African studies –e; there was only one West African study) among EVD patients. (DOCX 33 kb)

## Abbreviation

EVD: Ebolavirus disease
