# Supplementary material for: The face of Ebola: changing frequency of haemorrhage in the West African compared with Eastern-Central African outbreaks
Source: BMC Infect Dis. 2015 Dec 11;15:564. doi: 10.1186/s12879-015-1302-4 (PMC4676861; doi:10.1186/s12879-015-1302-4)
Supplement: Additional file 1: Table S1. — Number of EVD patients (note that in some studies the number of patients considered for the three bleeding features were different, suggesting that in some cases the clinical examination protocol was not the same within the study). (DOCX 14 kb) [file 12879_2015_1302_MOESM1_ESM.docx]

**Additional file 1: Table S1**

Number of EVD patients (note that in some studies the number of patients considered for the three bleeding features were different, suggesting that in some cases the clinical examination protocol was not the same within the study).

| Excluded study | Conjunctival bleeding | Nasal bleeding | Gingival bleeding |
| --- | --- | --- | --- |
|  |  |  |  |
| WHO, 1978 | 242 |  | 242 |
| Baron et al., 1983 |  | 32 | 32 |
| Sureau, 1989 |  | 265 | 265 |
| Bwaka et al., 1999 | 103 | 103 | 103 |
| Georges et al., 1999 | 15 | 15 | 15 |
| Khan et al., 1999 | 210 |  | 208 |
| Ndambi et al., 1999 | 23 | 23 | 23 |
| Mupere et al., 2001 | 20 | 20 | 20 |
| Roddy et al., 2012 | 26 | 26 | 26 |
| Maganga et al., 2014 | 38 | 38 | 38 |
| Schieffelin et al., 2014 | 44 |  |  |
| Bah et al., 2015 | 37 | 37 |  |
| Dallatomasina et al., 2015 | 245 |  |  |
| WHO Ebola Response Team, 2015 | 3756 | 3300 | 3306 |
| Yan et al., 2015 | 108 |  |  |
|  |  |  |  |
| Central African outbreaks | 677 | 522 | 972 |
| West Africa outbreak | 4190 | 3337 | 3306 |
| Overall | 4867 | 3859 | 4278 |
