# Supplementary material for: The face of Ebola: changing frequency of haemorrhage in the West African compared with Eastern-Central African outbreaks
Source: BMC Infect Dis. 2015 Dec 11;15:564. doi: 10.1186/s12879-015-1302-4 (PMC4676861; doi:10.1186/s12879-015-1302-4)
Supplement: Additional file 2: Figure S1. — Forest plots of the meta-analyses of the relative frequencies of conjunctival (a), nasal (b) and gingival (c) bleeding among EVD patients. Note the differences between Central and West African (Schieffelin et al., [54]; Bah et al., [55]; Dallatomasina et al., [56]; WHO, 2015 [57]; Yan et al., [58]) studies. (DOCX 27 kb) [file 12879_2015_1302_MOESM2_ESM.docx]

**Additional file 2: Figure S1**

Forest plots of the meta-analyses of the relative frequencies of conjunctival (a), nasal (b) and gingival (c) bleeding among EVD patients. Note the differences between Central and West African (Schieffelin et al., [54]; Bah et al., [55]; Dallatomasina et al., [56]; WHO, 2015 [57]; Yan et al., [58]) studies.

a

b

c
