# Supplementary material for: The face of Ebola: changing frequency of haemorrhage in the West African compared with Eastern-Central African outbreaks
Source: BMC Infect Dis. 2015 Dec 11;15:564. doi: 10.1186/s12879-015-1302-4 (PMC4676861; doi:10.1186/s12879-015-1302-4)
Supplement: Additional file 3: Table S2. — Sensitivity analysis to study inclusion. One study was excluded in turn and the pooled relative frequencies were re-estimated with the random-effects method. (DOCX 14 kb) [file 12879_2015_1302_MOESM3_ESM.docx]

**Additional file 3: Table S2**

Sensitivity analysis to study inclusion. One study was excluded in turn and the pooled relative frequencies were re-estimated with the random-effects method.

| Excluded study | Conjunctival bleeding and conjunctivitis | Nasal bleeding | Gingival bleeding |
| --- | --- | --- | --- |
|  |  |  |  |
| None | 33.6% (22.7-45.5%) | 8.7% (3.5-16.0%) | 21.1% (7.0-40.1%) |
|  |  |  |  |
| WHO, 1978 | 31.4% (20.7-43.3%) |  | 16.9% (7.3-29.4%) |
| Baron et al., 1983 |  | 7.5% (2.7-14.5%) | 19.9% (5.7-39.7%) |
| Sureau, 1989 |  | 7.7% (3.3-13.8%) | 21.0% (5.4-43.1%) |
| Bwaka et al., 1999 | 32.8% (21.2-45.6%) | 9.9% (3.6-18.8%) | 22.0% (6.5-43.3%) |
| Georges et al., 1999 | 32.2% (21.1-44.5%) | 8.2% (3.0-15.7%) | 19.5% (5.6-39.1%) |
| Khan et al., 1999 | 33.5% (21.2-47.2%) |  | 21.0% (5.7-42.6%) |
| Ndambi et al., 1999 | 30.1% (19.5-41.2%) | 9.0% (3.4-16.9%) | 20.2% (5.9-40.1%) |
| Mupere et al., 2001 | 33.1% (21.7-45.6%) | 8.5% (3.1-16.1%) | 22.1% (7.0-42.5%) |
| Roddy et al., 2012 | 32.3% (21.1-44.7%) | 8.7% (3.2-16.5%) | 23.0% (7.6-43.7%) |
| Maganga et al., 2014 | 35.3% (23.6-48.0%) | 8.4% (3.1-16.1%) | 22.5% (7.2-43.3%)) |
| Schieffelin et al., 2014 | 34.4% (22.7-47.2%) |  |  |
| Bah et al., 2015 | 35.9% (24.1-48.6%) | 9.0% (3.4-17.0%) |  |
| Dallatomasina et al., 2015 | 37.9% (28.3-48.0%) |  |  |
| WHO Ebola Response Team, 2015 | 34.9% (19.0-52.7%) | 10.1% (5.7-15.6%) | 24.2% (11.9-39.2%) |
| Yan et al., 2015 | 33.6% (22.7-45.5%) |  |  |
