# Supplementary material for: The face of Ebola: changing frequency of haemorrhage in the West African compared with Eastern-Central African outbreaks
Source: BMC Infect Dis. 2015 Dec 11;15:564. doi: 10.1186/s12879-015-1302-4 (PMC4676861; doi:10.1186/s12879-015-1302-4)
Supplement: Additional file 4: Figure S2. — Forest plots of the meta-analyses of the relative frequencies of conjunctival (Central African studies –a, West African studies –b), nasal (Central African studies –c, West African studies –d) and gingival bleeding (Central African studies –e; there was only one West African study) among EVD patients. (DOCX 33 kb) [file 12879_2015_1302_MOESM4_ESM.docx]

**Additional file 4: Figure S2**

Forest plots of the meta-analyses of the relative frequencies of conjunctival (Central African studies –a, West African studies –b), nasal (Central African studies –c, West African studies –d) and gingival bleeding (Central African studies –e; there was only one West African study) among EVD patients.

a

b

c

d

e
